# Psychopathology and cognitive performance in individuals with membrane-associated guanylate kinase mutations: a functional network phenotyping study

**DOI:** 10.1186/s11689-015-9105-x

**Published:** 2015-02-27

**Authors:** Kate Baker, Gaia Scerif, Duncan E Astle, Paul C Fletcher, F Lucy Raymond

**Affiliations:** Department of Medical Genetics, Cambridge Institute for Medical Research, University of Cambridge, Hills Road, Cambridge, CB2 0XY UK; Department of Experimental Psychology, University of Oxford, 9 South Parks Road, Oxford, OX1 3UD UK; MRC Cognition and Brain Sciences Unit, 15 Chaucer Road, Cambridge, CB2 7EF UK; Department of Psychiatry, University of Cambridge, Herchel Smith Building for Brain & Mind Sciences, Forvie Site, Robinson Way, Cambridge, CB2 0SZ UK

**Keywords:** MAGUK, Intellectual disability, Genetics, Cognition, Psychiatric disorders, *DLG3*

## Abstract

**Background:**

Rare pathogenic variants in membrane-associated guanylate kinase (MAGUK) genes cause intellectual disability (ID) and have recently been associated with neuropsychiatric risk in the non-ID population. However, it is not known whether risk for psychiatric symptoms amongst individuals with ID due to MAGUK gene mutations is higher than expected for the degree of general intellectual impairment, nor whether specific cognitive differences are associated with disruption to this gene functional network.

**Methods:**

This study addresses these two questions via behavioural questionnaires and cognitive testing, applying quantitative methods previously validated in populations with ID. We compared males with X-linked ID caused by mutations in three MAGUK genes (*PAK3*, *DLG3*, *OPHN1*; *n* = 9) to males with ID caused by mutations in other X chromosome genes (*n* = 17). Non-parametric and parametric analyses were applied as appropriate to data.

**Results:**

Groups did not differ in age, global cognitive impairment, adaptive function or epilepsy prevalence. However, individuals with MAGUK gene mutations demonstrated significantly higher psychopathology risks, comprising elevated total problem behaviours, prominent hyperactivity and elevated scores on an autism screening checklist. Despite these overt difficulties, individuals in the MAGUK group performed more accurately than expected for age and intelligence quotient (IQ) on computerised tests of visual attention, convergent with mouse models of MAGUK loss-of-function.

**Conclusions:**

Our findings support a role for MAGUK genes in influencing cognitive parameters relevant to psychiatric risk. In addition to establishing clear patterns of impairment for this group, our findings highlight the importance of careful phenotyping after genetic diagnosis, showing that gene functional network disruptions can be associated with specific psychopathological risks and cognitive differences within the context of ID.

**Electronic supplementary material:**

The online version of this article (doi:10.1186/s11689-015-9105-x) contains supplementary material, which is available to authorized users.

## Background

### Intellectual disability and psychiatric illness - the importance of specific aetiology

Intellectual disability (ID) encompasses any disorder involving “significant limitations both in intellectual functioning and in adaptive behaviour [which] originates before the age of 18 years” [1]. Lifetime risk of psychiatric disorder for individuals with ID is considerably elevated relative to the non-ID population [2], but few factors have been identified which predict mental health outcomes within the ID population [3,4]. The aetiology of ID demonstrates extreme heterogeneity, and the extent to which variation in mental health outcomes might be explained by aetiology is not currently known. Recently, there has been increased interest in understanding ID-associated mental health risk from an aetiological perspective, because advanced genomic technologies are able to identify a cause in an increasing proportion of ID cases [5,6]. Genetic diagnosis provides new opportunities to delineate cause-specific characteristics including mental health risks and to study mechanisms mediating neuropsychiatric outcomes. Although many studies have described psychiatric and cognitive phenotypes in relatively frequent genetic causes of ID such as 22q11.2 deletion syndrome and Fragile X syndrome, there have been remarkably few comparative studies of psychopathology and cognitive function in rare single gene causes of ID. Such studies provide important prognostic information for clinicians and families and are the starting point for experimental studies of potential therapeutic relevance.

### The rationale for gene functional network phenotyping

Given the rapidly increasing number of individual genes associated with ID (>1,000) and the rarity of each individual genetic diagnosis [7], it is difficult to generate prognostic and mechanistic information on a gene-specific basis. This study set out to establish whether classification of cases according to the molecular functional network of the causative gene mutation, coupled with theoretically guided post-genomic phenotyping, might identify network-associated psychiatric risks and cognitive mechanisms, a key step towards linking genetic diagnosis to mental health and, ultimately, improving outcomes in ID.

The empirical basis for gene functional networks relevant to ID pathogenesis is multi-fold. Diverse biochemical and cellular pathways have been identified over many years via study of rare syndromic forms of ID, and well-established mechanisms range from metabolic disturbance to abnormal neural proliferation and migration [8,9]. More recently, bioinformatic analysis of the gene repertoire of ID-associated copy number variants (CNVs) has highlighted discrete interconnected molecular networks involved in synaptogenesis, neurotransmission, and plasticity [10-13]. Further evidence for pathway-specific enrichments has been contributed from correlation of CNV gene sets with neurodevelopmental phenotypes in mouse models [14], synaptic proteome analysis [15] and transcriptome co-expression analysis [16,17]. Now, large-scale application of next-generation sequencing is identifying rare sequence variants within specific gene sets, in particular synaptic receptor-associated genes, ion channel-associated genes and chromatin-associated genes, with considerable overlap between genes and networks implicated in ID, autism and schizophrenia [18-21]. Via these multiple approaches, the functional genomic landscape of neurodevelopmental disorders is rapidly being mapped. A crucial next step is to carry out detailed comparative phenotypic investigations of individuals with neurodevelopmental disorders of known monogenic origin, to establish whether there is any homogeneity within groups and difference between groups defined by the function of the causative gene. The current study provides a first exploration of the practicality and utility of this functional networks phenotyping approach, focusing on X-linked intellectual disability genes previously implicated in psychopathology risks and specific aspects of cognition.

### Membrane-associated guanylate kinase genes and psychiatric risk

The membrane-associated guanylate kinase (MAGUK) genes encode p21-activated kinases that interact with postsynaptic complexes and regulate actin polymerisation via RhoGTPase signalling, influencing dendritic spine stabilisation and receptor localisation [22] [see Additional file 1]. Via these convergent activity-dependent mechanisms, MAGUK proteins exert a regulatory influence on neurotransmission and synaptic plasticity [23]. Loss-of-function mutations in several MAGUK genes have been identified as recurrent rare causes of X-linked intellectual disability (XLID). A loss-of-function mutation in *OPHN1* was the first XLID-associated MAGUK variant [24], followed by mutations in *PAK3* [25], *DLG3* [26] and *CASK* [27] in a number of further families. Phenotypes attributed to MAGUK mutations based on individual case reports encompass non-syndromic ID with additional neuropsychiatric features such as aggression, disinhibition and psychosis [28,29]. More recently, copy number variants and *de novo* sequence variants involving autosomal MAGUK genes have been identified in individuals ascertained for psychiatric disorders rather than ID, especially schizophrenia [19,30-32]. Existing evidence is therefore suggestive of a disproportionate elevation in mental health problems associated with disruption to the MAGUK functional network.

### Membrane-associated guanylate kinase genes and cognitive performance

Beyond overt psychiatric symptomatology, if functional genetic networks influence mental health risk via discrete neurodevelopmental pathways, ensuing divergent patterns of cognitive performance may be identifiable over and above general features of ID. Specific learning and behavioural changes have previously been identified in MAGUK mouse models, providing support for this proposal and some specific predictions about cognitive performance in individuals with MAGUK mutations. *Ophn1* knockout mice showed increased spontaneous activity levels and novelty-driven hyperactivity [33], and *Dlg3* knockout mice demonstrated motivation-dependent impairment in spatial learning [34]. Despite these impairments, it has recently been reported that *Dlg3*-null mice demonstrate faster-than-wild-type learning in a two-choice discrimination paradigm and enhanced attentional selection on a five-choice visual reaction time test. To explain these surprisingly divergent observations, authors suggest “the *Dlg3* paralog restrains or attenuates a specific aspect of the cognitive repertoire” [35], p. 18. Hence, a mutation might have a negative impact on adaptive behavioural function whilst paradoxically being associated with enhanced cognitive performance. Such observations of specific preservations of, or even advantages in, performance are of great value in specifying the precise mechanisms by which disrupted genetic pathways may influence psychiatric risks.

### Aims of the current study

To date, there has been no confirmation that risk of psychiatric symptoms in individuals with ID due to MAGUK mutations exceeds expectation for level of global intellectual impairment. Nor has it been determined whether specific aspects of cognitive processing are influenced by MAGUK mutations in humans, as suggested by studies in mouse models. The current study sought to address both of these questions by comparing males with XLID arising from MAGUK mutations to males with XLID due to mutations in genes with other known molecular functions. Each group was carefully phenotyped according to psychopathology and behavioural characteristics and then using a series of non-verbal computerised tasks engaging cognitive processes of interest. We predicted that individuals with MAGUK mutations would show elevated psychopathology but preserved cognitive functions in key domains.

## Methods

### Ethics and recruitment

This study received approval from the Central Cambridge Research Ethics Committee (project reference 11/0330/EE). Participants were originally recruited via regional genetics centres to the Genetics of Learning Disability (GOLD) study (http://goldstudy.cimr.cam.ac.uk/), eligibility criteria being ID without known cause and family history consistent with X-linked inheritance. Participants eligible for the current study were males over the age of 6 years in whom a pathogenic variant in a published XLID gene had been identified within the GOLD study and reported back to the family via the referring clinician. There were no exclusion criteria relating to severity of cognitive impairment or comorbidities. Written informed consent for participation was obtained from parents/carers (<16 years) and from adult participants who demonstrated capacity. For adults lacking capacity to consent to the study, a consultee was appointed, in keeping with the Mental Capacity Act (2005).

### Allocation to functional network groups

Systematically collated data on protein functions, biological process involvement, adult human brain regional expression patterns and developmental human brain expression trajectories for all study genes are provided in Additional file 1. All MAGUK genes have been implicated in postsynaptic actin dynamics and receptor trafficking via multiple independent methodologies. In contrast, non-MAGUK XLID genes have a variety of known neurodevelopmental functions not including postsynaptic actin dynamics: presynaptic vesicle trafficking (*AP1S2*, *SYP*), vesicle acidification (*SLC9A6*), the ubiquitin-proteosome system of protein degradation and cell cycle regulation (*CUL4B*, *HUWE1*, *UBE2A*) and the Wnt-signalling pathway (*PTCHD1*). MAGUK genes show common patterns of adult human brain expression (maximal expression in neocortex and hippocampus, low expression in subcortical and cerebellar structures), whereas the majority of non-MAGUK genes show either ubiquitous expression or maximal expression in subcortical structures [36]. MAGUK genes demonstrate gradually increasing expression levels across the prenatal period followed by stable expression or postnatal decline, whereas non-MAGUK genes show diverse developmental expression patterns [37].

Nine individuals from five families were recruited to the MAGUK group, with loss-of-function mutations in *OPHN1* (*n* = 1, one family), *DLG3* (*n* = 3, two families) and *PAK3* (*n* = 5, two families). Seventeen individuals from nine families were recruited to the XLID comparison group, with sequence variants or single gene CNVs involving *AP1S2* (*n* = 3), *SLC9A6* (*n* = 2), *SYP* (*n* = 2), *CUL4B* (*n* = 5), *HUWE1* (*n* = 3), *UBE2A* (*n* = 1) and *PTCHD1* (*n* = 1). The proportion of singleton participants vs familial participants did not differ between groups (singleton participants: MAGUK group *n* = 2, other XLID *n* = 4; familial participants: MAGUK group *n* = 7; other XLID *n* = 13; chi-square 0.04, *P* = 0.9).

### Questionnaire measures

Questionnaire measures were selected to be appropriate for ID populations and the age range of study participants. Questionnaires were administered by the same investigator (KB) in the participant’s home and were completed by a close family member or professional carer with long-term knowledge of the participant’s medical history, abilities and difficulties. A standardised, structured medical history interview was conducted, followed by administration of the Vineland Adaptive Behaviour scales, survey interview form [38], age-appropriate Developmental Behaviour Checklists (DBCs, an ID-specific 107 item psychopathology checklist) [39,40] and age-appropriate short-form Conners attention deficit hyperactivity disorder (ADHD) checklists [41,42].

Participant scores on Vineland scales and Conners questionnaires were scaled according to published normative age-appropriate data. For the DBC, total problem behaviour centiles were derived from age-appropriate normative data, stratified by ID severity according to the Vineland Adaptive Behaviour Composite classification. Autism checklist items were extracted from the DBC according to published criteria [43].

Psychopathology domain factors on the DBC were previously derived in separate studies of paediatric and adult ID populations (DBC-P and DBC-A manuals). To compare psychopathology domain scores across all cases irrespective of age, item scores were first extracted according to age-appropriate factors and then combined into five domains (disruptive, anxiety, self-absorbed, social relating, depression) from thematically consistent paediatric or adult factors, using percentage items checked as a consistent metric.

### Cognitive testing

Participants who were able to follow simple verbal instructions completed the four subtests of the Wechsler Abbreviate Scales of Intelligence-II (WASI-II) [44] and a suite of computerised tasks developed to investigate attentional processing in low-ability groups [45]. Cognitive testing was possible for eight individuals from the MAGUK group (mutations in *DLG3*, *PAK3*) and nine individuals from the XLID group (mutations in *AP1S2*, *SYP*, *CUL4B*, *HUWE1*, *UBE2A*, *PTCHD1*). For technical reasons, one individual from the MAGUK group did not complete the Go/NoGo task. One individual from the MAGUK group and one individual from the XLID group also did not complete the Go/NoGo task because of difficulty maintaining engagement.

Computerised tasks are described in detail in Scerif et al. [45], and the testing procedure was kept as similar as possible to this published method. In brief, the visual attention task battery comprises four short games (analogous to continuous performance tests) with the same easy-to-discriminate visual stimuli (Gabor patches) and basic instruction (“catch the fish”) with reinforcing auditory feedback after correct responses. Stimuli are presented via ePrime 2.0 on a laptop with 17-in. screen and external speakers, and participant responses are recorded via a button box. Each task begins with slow practice trials followed by real-time practice and 1-min test blocks with 15 targets per block. Interstimulus interval was fixed at 300 ms, stimulus duration 50 ms. The games were presented in a fixed order interleaved with WASI-II tasks to maintain motivation. Game 1 (detection) tests the ability to detect and respond to target stimuli (high-contrast Gabor patches) within a train of central fixation points; game 2 (oddball) tests the ability to select and respond to target stimuli (high-contrast Gabor patches) within a train of similar non-target stimuli (lower contrast Gabor patches); game 3 (visual-auditory crossmodal) reiterates the oddball task but irrelevant auditory stimuli at low and high intensity are presented simultaneous to each visual stimulus; game 4 (Go/NoGo) reverts to the detection task with additional non-target distractor stimuli (higher frequency Gabor patches). For each task, three outcome measures were assessed: omission rate (1-responses within 3,000 ms of target stimulus), commission rate (false alarm rate, including anticipatory responses and responses within 100 ms of target stimulus onset) and target response reaction time (RT, within-subject median time between target presentation and response).

### Statistics

All measures were checked for normality of distributions (Kolmogorov-Smirnov test) prior to parametric or non-parametric analysis as appropriate. Demographic variables, DBC total problem behaviour scores, DBC autism checklist scores and Conner’s ADHD index scores were normally distributed. DBC domain scores (% items checked) and full-scale intelligence quotient (FSIQ) scores were not normally distributed. Attention task performance scores were normally distributed after adjustment for age and FSIQ. Age and FSIQ were included as covariates in analysis of variance for attention task performance and exploratory correlational analyses between cognitive and behavioural measures.

## Results

### Demographics

MAGUK and XLID comparison groups did not differ in age, severity of global intellectual impairment as estimated by Vineland Adaptive Behaviour Composite score, gross motor ability or history of epilepsy (Table 1). Groups did not differ in Vineland adaptive domain profiles (repeated measures ANOVA group effect *F*_1,23_ = 0.912, *P* = 0.35, group × domain interaction *F*_2,46_ = 0.76, *P* = 0.48). For both groups, communication skills were relatively more impaired than daily living skills and socialisation skills, which were in turn relatively more impaired than motor skills. In summary, gene functional network did not predict severity of intellectual disability or profile of adaptive impairments.

**Table 1:** **Study participants**

**Demographic measure**		**Gene functional group**		***P value***
		**MAGUK (** ***n*** **= 9)**	**XLID (** ***n*** **= 17)**	
Age at assessment/years	range	13.4 to 40.4	6.2 to 54	0.28
	mean (SD)	24.5 (9.2)	30.4 (14.3)	
Gross motor ability^a^	range	1 to 16	1 to 16	0.11
	mean (SD)	10.6 (5.0)	7.2 (4.7)	
Global intellectual ability^b^	range	20 to 61	20 to 67	0.40
	mean (SD)	44 (14)	38 (18)	
Mild or moderate impairment	% within functional group	66.7	41.2	0.22
Severe or profound impairment	% within functional group	33.3	58.8	
History of seizures	% within functional group	11.1	35.3	0.19

^a^Vineland age-appropriate standard score; ^b^Vineland Adaptive Behaviour Composite standard score, general population mean 100.

#### Psychopathology

**Figure 1:**
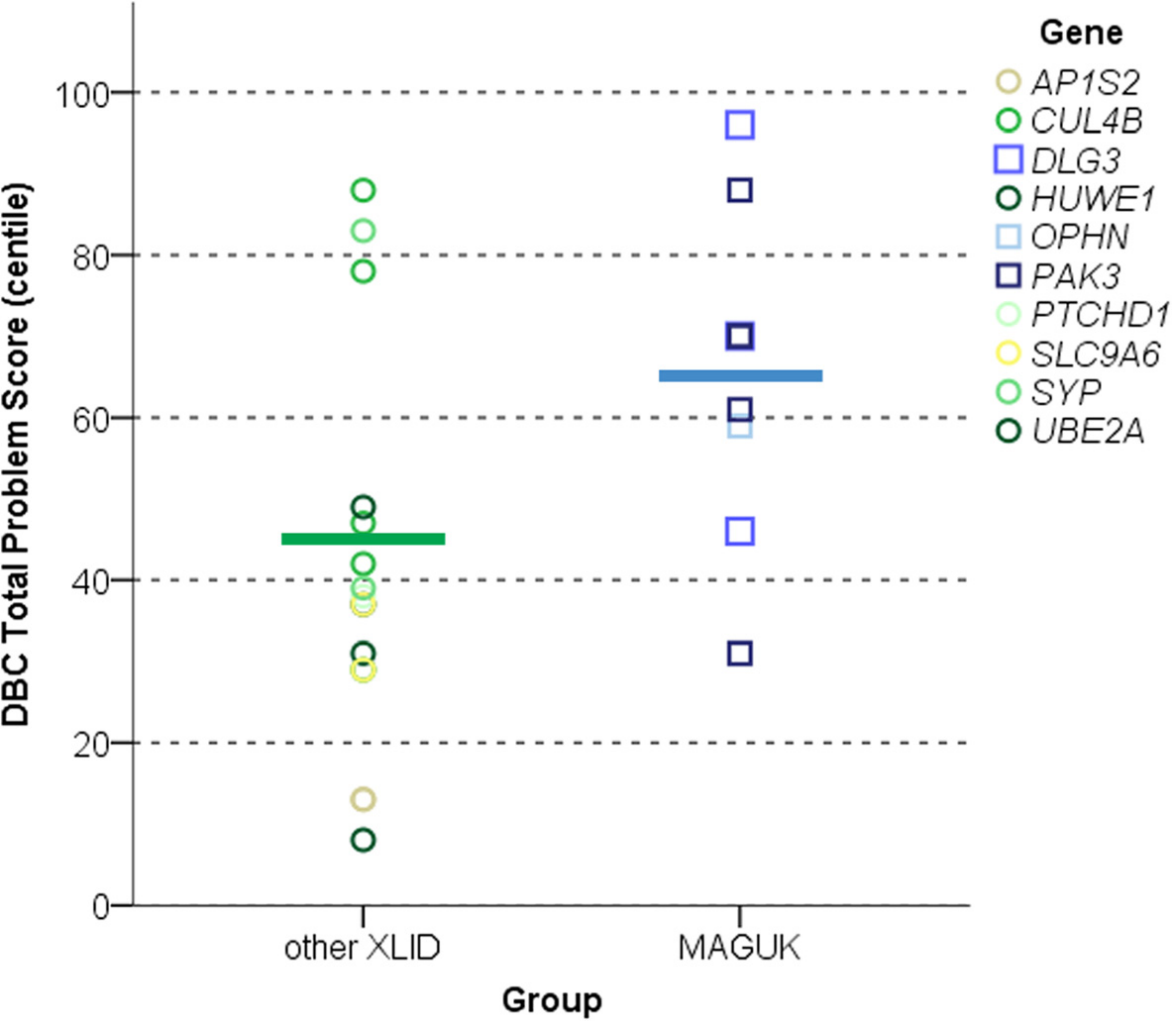
**Psychopathology summary scores in MAGUK cases and XLID controls.** Total problem behaviour scores for individual subjects labelled by causative gene mutation (Developmental Behaviour Checklists, age-stratified and ID-severity-stratified centiles). Participant data is grouped according to gene functional network - MAGUK (*n* = 9), XLID (*n* = 17). DBC, Developmental Behaviour Checklist; MAGUK, membrane-associated guanylate kinase; XLID, X-linked intellectual disability.

**Figure 2:**
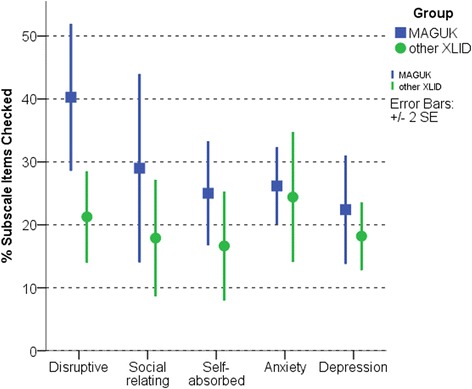
**Psychopathology domain scores in MAGUK cases and XLID controls.** Domain-specific problem behaviour scores (Developmental Behaviour Checklists, % items checked within manual-defined factors). Participant data is grouped according to gene functional network - MAGUK (*n* = 9), XLID (*n* = 17). MAGUK, membrane-associated guanylate kinase; XLID, X-linked intellectual disability, SE, standard error.

The MAGUK group had significantly higher DBC total problem behaviour centile scores stratified for age and ID severity (Table 2, Figure 1). Groups also differed in relative risks for specific emotional and behavioural difficulties assessed via the DBC (Figure 2). The MAGUK group demonstrated significantly higher scores in the disruptive domain (Mann-Whitney U *P* = 0.004), non-significant higher scores in the social-relating domain (*P* = 0.085) and no difference from the XLID control group in anxiety, self-absorbed or depression domains (all *P* > 0.2). The MAGUK group had higher DBC autism checklist scores and contained a higher proportion of individuals meeting screening criteria for a possible diagnosis of an autistic disorder (67% vs 18.8%, Pearson X^2^*P* = 0.017). Conner’s ADHD index T scores differed significantly between groups. Repeated measures ANOVA for inattention, hyperactivity and oppositional subscale T scores indicated a significant main effect of group (*F*_1,15_ = 8.52, *P* = 0.008) and a trend towards significant group × subscale interaction (*F*_2,30_ = 2.69, *P* = 0.08), with MAGUK cases differing from XLID controls in hyperactivity and inattention and not differing in oppositional behaviours (Table 2).

**Table 2:** **Psychopathology rating scales**

**Measure**		**Group**		***t (P value)*** ^***c***^
		**MAGUK (n = 9)**	**XLID (n = 17)**	
DBC total problem behaviour score^a^/centile	range	31 to 96	8 to 88	2.9 (0.009)
	mean (SD)	65.7 (19.8)	40.6 (23.3)	
DBC autism screening assessment^b^/raw score	range	12 to 30	2 to 28	2.4 (0.028)
	mean (SD)	17.8 (5.3)	11.9 (7.1)	
Conners ADHD index/T score	range	72 to 86	41 to 86	2.7 (0.004)
	mean (SD)	77.7 (5.5)	60.3 (15.1)	
Conners A (oppositional)/T score	mean (SD)	65.7 (18.1)	57.3 (10.8)	1.1 (0.33)
Conners B (inattention)/T score	mean (SD)	69.8 (6.85)	56.9 (11.2)	2.9 (0.01)
Conners C (hyperactivity) T score	mean (SD)	77.7 (16)	53.1 (15.1)	3.1 (0.01)

^a^Developmental Behaviour Checklists, adult or paediatric norms; ^b^Developmental Behaviour Checklists, paediatric manual scoring (all subjects); ^c^equal variances not assumed.

To assess whether observed differences between groups in psychopathology scores could be confounded by intelligence quotient (IQ), analysis was repeated comparing only MAGUK and XLID subjects for whom FSIQ estimates were available (see below for confirmation of IQ matching between subgroups who completed cognitive testing). Despite smaller participant numbers, results in this higher ability subsample were consistent with whole sample comparisons: MAGUK participants had significantly higher DBC total problem behaviour scores (*t* = 2.67, *P* = 0.018), higher Conners ADHD index scores (*t* = 2.83, *P* = 0.017) and higher DBC autism checklist scores (*t* = 3.04, *P* = 0.009).

To explore whether observed differences between groups in psychopathology scores could be confounded by age, data was inspected separately for participants under the age of 18 years (MAGUK *n* = 4, other XLID *n* = 4) and aged 18 years and over (MAGUK *n* = 5; other XLID *n* = 13). Differences between groups in problem behaviour scores were consistent in both cross-sectional samples. The difference in median DBC total problem behaviour scores was 30 centile points for the younger age group and 24 for the older age group. Similarly, median Conners ADHD index T-scores differed by 20 points for the younger age group and 17 for the older age group. A potential developmental effect was observed for DBC autism checklist scores, in that median checklist scores differed by only four points for the younger age group but nine points for the older age group.

#### Cognitive task performance

For the MAGUK subgroup able to participate in cognitive testing (*n* = 8), IQ estimated by the WASI-II ranged from 40 to 73 (mean 51.3, standard deviation (s.d.) 11.3). For the XLID subgroup able to participate in cognitive testing (*n* = 9), IQ ranged from 43 to 65 (mean 56.6, s.d. 8.6). IQ scores did not differ significantly between these MAGUK and XLID subgroups (Mann-Whitney U *P* = 0.3) who went on to complete computerised cognitive tasks.

**Figure 3:**
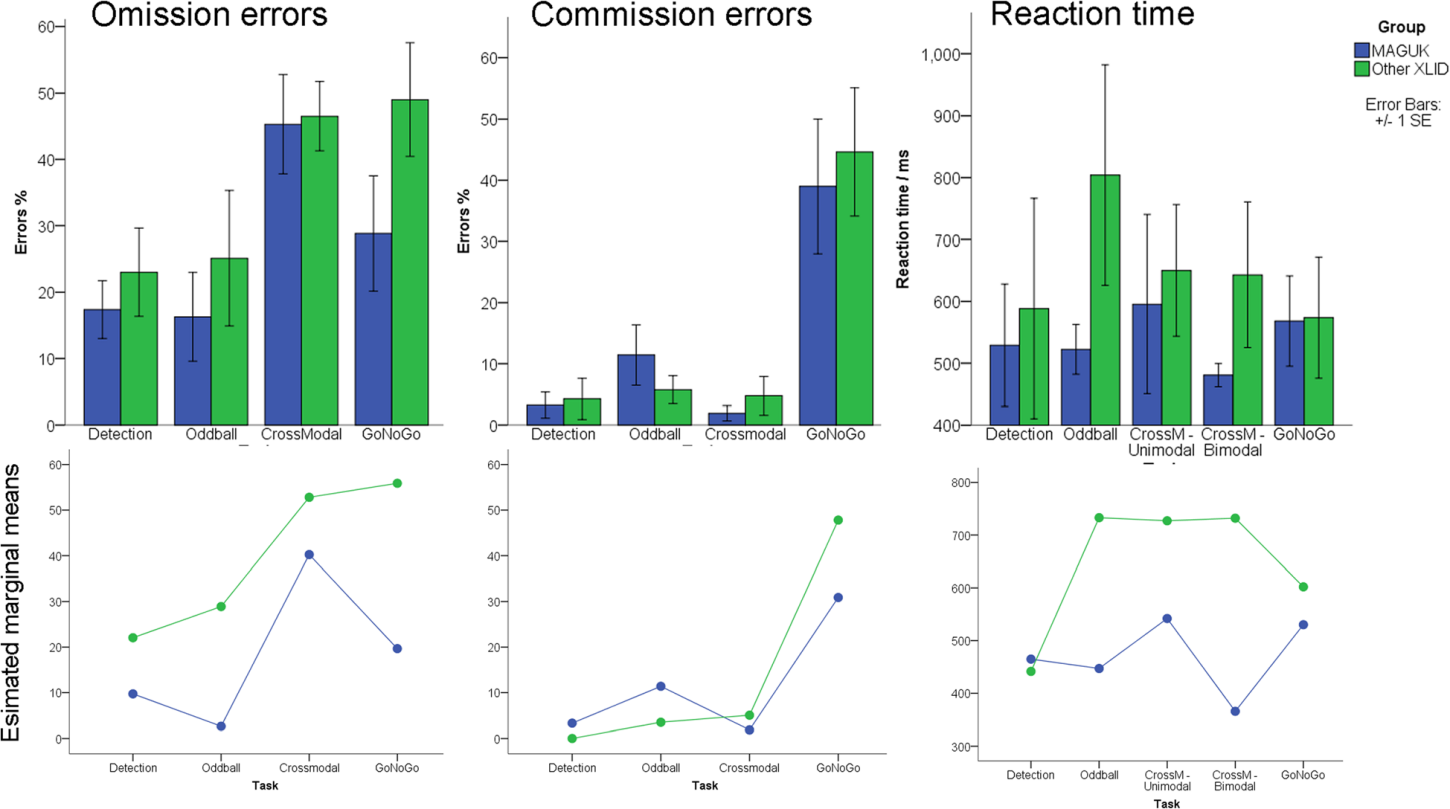
**Attention task performance in MAGUK cases and XLID controls.** Analysis of visual attention task parameters for MAGUK (*n* = 8) and XLID (*n* = 9) subjects who completed cognitive testing. Bar charts present raw error scores and reaction times (means, standard errors) for each of the four visual attention tasks. Line graphs present age-adjusted and IQ-adjusted marginal means (repeated measures ANOVA). MAGUK, membrane-associated guanylate kinase; XLID, X-linked intellectual disability, SE, standard error.

Group differences in three outcome measures (omission rate, commission rate and median RT for correct responses) were examined across the four visual attention tasks - detection, oddball, crossmodal, Go/NoGo (Figure 3). Omission rates were significantly lower in the MAGUK group (*F*_1,10_ = 5.46, *P* = 0.04), with no significant interaction between group and task (*F*_3,30_ = 2.2, *P* = 0.11). Commission rates were low across all tasks except the Go/NoGo task, and rates did not differ between groups (*F*_1,10_ = 0.10, *P* = 0.76), indicating that the higher accuracy of responding to targets amongst the MAGUK group was not explained by increased numbers of random button presses or impulsive responding. Raw data suggested that the MAGUK group responded to targets on average faster than XLID controls, but repeated measures ANOVA covarying for age and IQ did not detect significant difference (*F*_1,10_ = 2.49, *P* = 0.15), with no significant group × task interaction (*F*_3,30_ = 1.24, *P* = 0.3). Hence, there was no evidence that the relatively strong behavioural performance demonstrated by MAGUK individuals was attributable to the groups using different speed-accuracy trade-offs. Indeed, the trend towards faster responses in the MAGUK group demonstrates the opposite.

#### Association between psychopathology scores and attention task performance

Exploratory correlational analysis was carried out to identify potential associations between cognitive parameters and behavioural disturbance within the two study groups. Analysis was limited to factors with the greatest between-groups effect sizes (DBC disruptive domain score, DBC autism screening checklist score, visual oddball omission error rate and RT). Partial correlations covarying for age and IQ suggest that within the XLID control group, poor performance on visual attention tasks may be associated with increased behavioural symptoms: oddball omission error rate was weakly associated with DBC disruptive score (*R* = 0.66, *P* = 0.11, Cohen’s *d* = 1.76) and autism checklist score (*R* = 0.58, *P* = 0.23, Cohen’s *d* = 1.42); no associations between oddball RT and either behavioural score were observed. For the MAGUK group, a contrasting pattern may be present, whereby a relatively strong performance on visual attention tasks may be associated with increased autism symptoms: omission error rate (on average low within this group) was not associated with either DBC problem behaviour scores or autism checklist scores; a significant association between fast oddball RT and high scores on the autism checklist was observed (*R* = −0.92, *P* = 0.027, Cohen’s *d* = 4.69), whilst no association between RT and DBC disruptive scores was observed (*R* = −0.38, *P* = 0.5, Cohen’s *d* = 0.8).

## Discussion

In this study, we sought evidence that the functional network of causative gene mutations could predict aspects of ID phenotype. Specifically, we tested the hypothesis that mutations in MAGUK genes, implicated in psychiatric disorders in the non-ID population, would be associated with rates of psychopathology above expectation for level of global functioning. This hypothesis was supported - gene function did not predict severity of intellectual impairment (assessed via a questionnaire measure of adaptive function for the whole sample, as well as IQ testing for individuals with mild-to-moderate ID), but did predict behavioural problems. Moreover, we uncovered evidence for specific cognitive processing differences between gene functional network groups, yielding new hypotheses for post-genomic research into the mechanisms of psychopathology risk.

### Psychopathology risks associated with MAGUK mutations

Frequently reported symptoms amongst males with MAGUK mutations were hyperactivity, inattention and social interaction difficulties contributing to high autism screening scores. Although these problems were not unique to the MAGUK group, they were remarkably consistent across individuals and families with different MAGUK gene mutations, irrespective of ID severity. Symptoms were reported to have current impact on day-to-day function for participants of all ages, indicating a persistent negative effect of these mutations on emotional and social functioning, rather than delay in behavioural maturation. Problem behaviours reported for individuals with MAGUK mutations tended to be comorbid across several domains, typified by high scores on inattention scales and autism scales. The mechanisms underlying psychopathology risk in this population do not appear to respect psychiatric diagnostic categories, in common with other high-risk groups identified on a genomic basis [46].

Our observations yield further hypotheses regarding the neurobiological and developmental origins of psychiatric symptoms within this aetiologically defined group. Disrupted structural and functional connectivity within frontostriatal systems impacting upon cognitive control has previously been implicated in the co-occurrence of autism and inattention in a subset of individuals with neurodevelopmental disorders of unknown aetiology [47,48]. Defining the neurobiological basis for psychiatric symptoms within the MAGUK population could therefore contribute to models of heterogeneity and comorbidity within the behaviourally defined populations of autism and ADHD.

### Cognitive processing differences associated with MAGUK mutations

Despite overt symptoms including inattention and hyperactivity, the MAGUK group demonstrated better target detection accuracy than the XLID comparison subjects across four visual attention tasks. This observation is convergent with the previous observation of better-than-wild-type performance on visual discrimination and selective response tasks in mice with loss-of-function *Dlg3* mutations [35]. Whilst better-than-control task performance in association with comorbid ID and psychiatric symptoms may be counter-intuitive, it is not unprecedented. Enhanced visual discrimination and atypical parameters of selective attention have previously been observed in some individuals with autism [49-51]. This is intriguing given that individuals within the MAGUK group had both higher scores on an autism screening checklist and more accurate visual attention performance than XLID controls, and that speed of responses was associated with autism symptoms within the MAGUK group. To date, studies of visual processing in autism have concentrated on high-functioning individuals without ID, and we are not aware of another study that has observed atypically enhanced visual attention functions in individuals with intellectual disability plus autism spectrum traits. Our observations lend support to a developmental model whereby atypical perceptual processing and attentional control contribute to the emergence of social cognitive impairments, irrespective of global cognitive ability.

One explanation for unexpectedly accurate visual target detection in MAGUK-associated ID could be that this cognitive bias is a ubiquitous characteristic of autism, with no relationship to aetiology. However, a previous study applying the identical cognitive testing protocol does not support this explanation - cognitive performance advantages for the MAGUK group contrasts with impaired performance on the same tasks in children with Fragile X syndrome [45], despite similar IQ range and behavioural characteristics including inattention and autism spectrum disorders [52]. This contrast could be explained by methodological differences between the studies, in particular the older age of the MAGUK subjects and higher IQ of control subjects in the Fragile X study. However, an alternative explanation is that distinct neurodevelopmental pathways may mediate psychopathology risks in these two ID populations. It has recently been shown that a dominant negative inhibitor of PAK3 induced hyperactivity and stereotypy in WT mice and ameliorated these phenotypes to WT levels in FMR1 knockout mice [53]. Hence, although the behavioural phenotypes in males with MAGUK mutations and Fragile X syndrome are similar, disruptions to mGluR-dependent dendritic biology and cognitive correlates may be directly opposite. More generally, divergent results in the MAGUK and Fragile X studies suggest that investigating cognitive processes within ID populations on an aetiology-first basis may be informative of heterogeneous mechanisms underlying developmental psychopathology.

### Limitations

The results of the current study are limited by the rarity of MAGUK mutations and the small numbers of diagnosed individuals available for recruitment, not encompassing all MAGUK genes associated with ID, limiting statistical power and generalisability of findings. A number of potential confounding factors, including family status (sibling versus isolated cases) and severity of biochemical disruption predicted by individual mutations, cannot be factored into analysis without a much larger dataset and application of more complex statistical modelling. Specific genetic factors influence neurodevelopment within defined time periods, from embryological development to early and later postnatal life. Each functional network may impact differentially on each phase of neurodevelopment; hence, a developmental approach to investigation of phenotypes and mechanisms, ideally investigated longitudinally, would be highly desirable once a sufficient number of cases have been diagnosed. This approach has already been successful in pinpointing dynamic developmental changes in phenotypes within and between ID-associated syndromes [54,55]. The current study compares average phenotypic characteristics across a relatively wide age range of participants and does not include young children or infants who may be informative of divergent trajectories of neurodevelopmental impairment. These are necessary future steps for understanding complex neurodevelopmental disorders of genetic origin.

Nevertheless, this is the largest study to date of individuals with ID due to MAGUK mutations and the only study to have adopted systematic methodologies including assessment of comparison subjects with ID of known genetic aetiology. We observed consistencies across measures (both psychopathological and cognitive), with elevated symptoms on independent questionnaire measures and superior target-detection accuracy on four visual attention tasks. It was beyond the scope of this project to conduct diagnostic psychiatric evaluations; however, concordance between psychopathology questionnaires applied in the study and clinician diagnosis has previously been established [39,56]. On the basis of the questionnaire data we have collected and comparative differences between groups in autism checklist scores, comprehensive investigation of autism symptoms and diagnoses using an ID-appropriate standardised assessment would be desirable in a future study of this population. Similar considerations apply to a more detailed and sensitive assessment of verbal and non-verbal intellectual abilities across the full range of adaptive function.

This study established that it was possible to collect cognitive test data from the majority of participants with ID. This is encouraging, given the need for detailed phenotyping beyond IQ testing and psychopathology rating scales. To achieve these goals, a host of theory-driven and experimentally valid measures are required to quantify parameters of cognitive function in populations with ID via behavioural and neurophysiological methods. Many standardised assessment batteries result in floor-level performance and describe domains of disability, rather than quantifying cognitive performance variables within the ability range of subjects which may be informative of developmental mechanisms. If and when large-scale multi-dimensional phenotyping datasets can be acquired, it will be possible to apply statistical approaches, for example topological modelling, to identify genome-phenome associations without pre-specifying genes and networks of interest. Identification of these associations can in turn yield novel hypotheses and stimulate interdisciplinary investigation into the neurobiological mechanisms underlying the diversity of intellectual disability.

## Conclusions

In this study, ID-causing mutations within the MAGUK functional network were found to be associated with an elevated risk of psychiatric disturbance across several symptom domains. We also found preliminary evidence for a relationship between symptom risk and specific cognitive parameters, consistent with previous animal studies. Our data provides first evidence in humans for a model in which MAGUK mutations, which influence the dynamic organisation of postsynaptic actin scaffolding, may alter short-term plasticity to enhance aspects of attentional processing to the detriment of social and behavioural development. Further integrative studies are required to test, extend and elaborate this model, which may be relevant to the developmental evolution of psychiatric symptoms in individuals with and without ID. More generally, these results confirm that genetic aetiology contributes to variability in mental health outcomes within the ID population. We have confirmed that it is possible and informative to investigate psychiatric and cognitive heterogeneity associated with rare genetic causes of ID, classified from the perspective of functional gene networks.

## Additional file

Additional file 1: Table S1. Evidence for functional network classification of MAGUK and non-MAGUK X-linked Intellectual Disability genes. Descriptive data on biochemical functions, biological process involvement, human brain expression (adult, developmental) and signalling pathway involvement.

## Abbreviations

ADHD: attention deficit hyperactivity disorder
CNV: copy number variant
DBCs: Developmental Behaviour Checklists
FSIQ: full-scale intelligence quotient
GOLD: Genetics of Learning Disability study
ID: intellectual disability
IQ: intelligence quotient
MAGUK: membrane-associated guanylate kinase
RT: reaction time
XLID: X-linked intellectual disability
