# Supplementary material for: Psychopathology and cognitive performance in individuals with membrane-associated guanylate kinase mutations: a functional network phenotyping study
Source: J Neurodev Disord. 2015 Feb 27;7(1):8. doi: 10.1186/s11689-015-9105-x (PMC4369839; doi:10.1186/s11689-015-9105-x)
Supplement: Additional file 1: Table S1. — Evidence for functional network classification of MAGUK and non-MAGUK X-linked Intellectual Disability genes. Descriptive data on biochemical functions, biological process involvement, human brain expression (adult, developmental) and signalling pathway involvement. [file 11689_2015_9105_MOESM1_ESM.docx]

Table 1: Evidence for functional network classification of MAGUK and non-MAGUK X-linked Intellectual Disability genes

| **Gene** | **Protein** | **Biochemical function** | **Molecular processes** | **Biological processes** | **Human adult brain expression** | **Human developmental brain expression** | **Signalling pathway involvement** |
| --- | --- | --- | --- | --- | --- | --- | --- |
| **Resource** | **ncbi.nlm.nih.gov/omim** | **genecards.org** | **geneontology.org** | **geneontology.org** | **braineac.org** | **hbatlas.org** | **genome.jp/kegg** |
| ***AP1S2*** | Adaptor-Related Protein Complex 1, Sigma 2 Subunit | Protein sorting in the late-Golgi/trans-Golgi network and endosomes. | Protein transporter activity | Intracellular protein transport, vesicle-mediated transport | Max – medulla, white matter  Min – cerebellum, thalamus | Stable prenatal and postnatal | Lysosome |
| ***CUL4B*** | Cullin 4b | Component of multiple cullin-RING-based E3 ubiquitin-protein ligase complexes which mediate ubiquitination and proteasomal degradation of target proteins. | Ubiquitin protein ligase binding | DNA repair, cell cycle regulation | Brain-wide, no variation | Maximum early prenatal. Stable postnatal | Nucleotide excision repair, ubiquitin-mediated proteolysis |
| ***DLG3*** | Discs, Large Homolog 3 | Membrane-associated guanylate kinase | Guanylate kinase activity, ionotropic glutamate receptor binding, PDZ domain binding | Establishment of planar polarity, synaptic transmission, axon guidance | Max – hippocampus, cortex  Min – medulla, white matter | Increases across prenatal, declines from late childhood | Hippo signalling |
| ***HUWE1*** | HECT, UBA and WWE domain containing 1 | E3 ubiquitin-protein ligase which mediates ubiquitination and proteasomal degradation of target proteins. | DNA binding, ubiquitin-protein ligase activity | Base-excision repair, protein, ubiquitination, histone ubiquitination | Brain-wide, no variation | Stable prenatal and postnatal | Ubiquitin mediated proteolysis |
| ***OPHN1*** | Oligophrenin 1 | Rho-GTPase-activating kinase that promotes GTP hydrolysis of Rho subfamily members | Rho GTPase activator activity, phospholipid binding, ionotropic glutamate receptor binding, actin binding, | Cell migration, cell extension,  small GTPase mediated signal transduction, axon guidance, actin cytoskeleton organization,  regulation of endocytosis, regulation of synaptic transmission, glutamatergic | Max – hippocampus  Min - cerebellum | Increases during prenatal life. Stable postnatal | No hits |
| **Gene** | **Protein** | **Biochemical function** | **Molecular processes** | **Biological processes** | **Human adult brain expression** | **Human developmental brain expression** | **Signalling pathway involvement** |
| **Resource** | **ncbi.nlm.nih.gov/omim** | **genecards.org** | **geneontology.org** | **geneontology.org** | **braineac.org** | **hbatlas.org** | **genome.jp/kegg** |
| ***PAK3*** | p21 protein (Cdc42/Rac)-Activated Kinase 3 | Serine/threonine protein kinase.  Acts as downstream effector of small GTPases | Rho GTPase binding, protein tyrosine kinase activity, receptor signalling protein serine/threonine kinase activity | MAPK cascade  activity, NOT mitotic cell cycle, protein phosphorylation, actin filament organization, axonogenesis, dendrite development, synapse organization, dendritic spine morphogenesis | Max – hippocampus, cortex  Min – cerebellum, white matter | Increases during prenatal life. Stable postnatal (postnatal decline in cerebellum) | Axon guidance, ErbB signalling, Ras signalling, focal adhesion, regulation of actin cytoskeleton |
| ***PTCHD1*** | Patched domain containing 1 | Membrane protein with a patched domain, similar to Drosophila proteins which act as receptors for the morphogen sonic hedgehog | Hedgehog receptor activity | Smoothened signalling pathway | Max – cerebellum  Min – hippocampus, white matter | Regionally-specific prenatal expression patterns. Marked postnatal increase in cerebellum, other regions stable. | No hits |
| ***SLC9A6*** | Solute carrier family 9, subfamily A (NHE6, cation proton antiporter 6), member 6 | Electroneutral exchange of protons for Na(+) and K(+) across the early and recycling endosome membranes. | Sodium:hydrogen antiporter activity | Regulation of pH , sodium ion transmembrane transport | Max – cortex  Min – cerebellum, white matter | Maximum late prenatal, slight postnatal decline | Cardiac muscle contraction |
| ***SYP*** | Synaptophysin | Integral membrane protein of small synaptic vesicles in brain and endocrine cells. Directs targeting of vesicle-associated membrane protein 2 (synaptobrevin) to intracellular compartments | Transporter activity , calcium ion binding, protein binding, cholesterol binding, syntaxin-1 binding | Endocytosis,  synaptic vesicle maturation,  regulation of neuronal synaptic plasticity, regulation of opioid receptor signalling pathway | Max - cortex  Min – white matter | Marked prenatal increase, stable postnatal | SNARE interactions in vesicular transport |
| ***UBE2A*** | Ubiquitin-Conjugating Enzyme E2A | Plays a role in transcription regulation by catalyzing the monoubiquitination of histone H2B | Ubiquitin-protein ligase activity | DNA repair, ubiquitin-dependent catabolic process , regulation of cell proliferation, response to UV  histone H2A ubiquitination | Max – white matter  Min – cortex, cerebellum | Stable prenatal and postnatal | Ubiquitin mediated proteolysis |
